# Mitochondria-targeted antioxidant protects against irradiation-induced salivary gland hypofunction

**DOI:** 10.1038/s41598-021-86927-3

**Published:** 2021-04-08

**Authors:** Xibao Liu, Krishna P. Subedi, Changyu Zheng, Indu Ambudkar

**Affiliations:** 1 Secretory Physiology Section, National Institute of Dental and Craniofacial Research, NIH, 9000 Rockville Pike, Bethesda, MD 20892 USA; 2 NIH, Building 10, Room 1N-113, Bethesda, MD 20892 USA

**Keywords:** Calcium signalling, Ion channel signalling

## Abstract

A severe consequence of radiation therapy in patients with head and neck cancer is persistent salivary gland hypofunction which causes xerostomia and oral infections. We previously showed that irradiation (IR) of salivary glands in mice triggers initial transient increases in mitochondrial reactive oxygen species (ROS_mt_), mitochondrial [Ca^2+^] ([Ca^2+^]_mt_), and activated caspase-3 in acinar cells. In contrast, loss of salivary secretion is persistent. Herein we assessed the role of ROS_mt_ in radiation-induced irreversible loss of salivary gland function. We report that treatment of mice with the mitochondrial-targeted antioxidant, MitoTEMPO, resulted in almost complete protection of salivary gland secretion following either single (15 Gy) or fractionated (5 × 3 Gy) doses of irradiation. Salivary gland cells isolated from MitoTEMPO-treated, irradiated, mice displayed significant attenuation of the initial increases in ROS_mt_, ([Ca^2+^]_mt_, and activated caspase-3 as compared to cells from irradiated, but untreated, animals. Importantly, MitoTEMPO treatment prevented radiation-induced decrease in STIM1, consequently protecting store-operated Ca^2+^ entry which is critical for saliva secretion. Together, these findings identify the initial increase in ROS_mt_, that is induced by irradiation, as a critical driver of persistent salivary gland hypofunction. We suggest that the mitochondrially targeted antioxidant, MitoTEMPO, can be potentially important in preventing IR-induced salivary gland dysfunction.

## Introduction

Neurotransmitter-stimulated increase in cytosolic [Ca^2+^], [Ca^2+^]_i_, in acinar cells is the primary trigger for fluid secretion from salivary glands^1,2^. This is achieved by receptor-dependent activation of phospholipase C which cleaves plasma membrane lipid phosphatidyl inositol 4,5 bisphosphate (PIP_2_) and causes generation of inositol trisphosphate (IP_3_), IP_3_-mediated release of Ca^2+^ from the endoplasmic reticulum and activation of Ca^2+^ entry. The latter is essential for maintaining a sustained elevation of [Ca^2+^]_i_ which drives salivary fluid secretion. This Ca^2+^ entry is dependent on the Store-Operated Ca^2+^ Entry (SOCE) mechanism which is activated when ER-[Ca^2+^] is decreased due to IP_3_-mediated release of Ca^2+^ from the ER stores. ER Ca^2+^-sensor proteins, STIM1 and STIM2, sense the decrease in ER-[Ca^2+^] and cluster in the periphery of the cells where they recruit and activate Orai1 channels that mediate SOCE^1–3^. Aberrant SOCE has been linked with decrease in salivary gland function associated with the autoimmune exocrinopathy in Sjogren’s Syndrome mouse models^4,5^ as well as following radiation to salivary glands in mice^6^.

A devastating side effect of irradiation (IR) in patients receiving radiation treatment for head and neck cancers is xerostomia, or dry mouth, a symptom of severe decrease in saliva secretion^7–10^ which is often accompanied by problems with eating and swallowing food as well as oral infections. Together these issues severely affect the quality of life of the patient. Currently, there are no well-established procedures that either prevent IR-induced loss of salivary gland function or induce recovery of the function post-IR. Furthermore, the exact mechanism(s) involved in IR-induced salivary gland dysfunction is poorly understood. Consequences of ionizing radiation in any tissue is DNA damage as well as substantial increase in cellular levels of reactive oxygen species (ROS)^11^. An increase in ROS has been implicated as a major cause of pathology in a number of tissues^12^. Our previous studies have shown that IR of salivary glands targets a ROS sensing Ca^2+^ channel, transient receptor potential melastatin-like 2 (TRPM2), in the plasma membrane and that ROS-dependent activation of Ca^2+^ entry via TRPM2 is linked with loss of salivary flow. Mice lacking TRPM2 show a transient loss of saliva secretion with recovery of more than 80% of the function within a month after IR^13^. Interestingly, TRPM2 remains active only for a relatively short time after IR, while loss of salivary function is persistent^6,13^. Subsequently, we identified early effects of IR on the mitochondria, including increases in mitochondrial [Ca^2+^] ([Ca^2+^]_mt_), mitochondrial ROS (ROS_mt_), and loss of membrane potential that are detected within 24 h and last for about 10 days. Our findings revealed an amplification loop whereby an increase in plasma membrane Ca^2+^ permeability through TRPM2 triggers accumulation of Ca^2+^ in the mitochondria and enhancement of mitochondrial ROS. ROS generated in mitochondria and ADPR, produced locally within the organelle, are released into the cytosol and lead to amplification of TRPM2 activity^6^. Further, these mitochondrial changes induced activation of caspase-3 which caused a decrease in the ER protein STIM1 and consequently loss of SOCE in acinar cells. Together, our previous data demonstrated that mitochondria are early responders and central players in IR-induced salivary gland dysfunction. However, these findings did not elucidate the exact change(s) in mitochondria that drive IR-induced salivary gland dysfunction.

The current study was designed to test the role of ROS_mt_ in radiation-induced salivary gland hypofunction. Towards this, we developed a protocol for treating mice with the mitochondrially targeted ROS scavenger, MitoTEMPO, and tested its effect in IR-induced loss of salivary gland function. Herein, we describe our novel findings which reveal that MitoTEMPO treatment provides significant protection of salivary gland function following either single (15 Gy) or fractionated (5 × 3 Gy) irradiation of mice salivary glands. We show that IR-induced initial increase in ROS_mt_ and [Ca^2+^]_mt_, as well as level of activated caspases 3, in salivary gland acinar cells are attenuated in MitoTEMPO-treated mice as compared to those that were irradiated but did not receive the ROS scavenger. Importantly, STIM1 protein as well as SOCE are preserved after IR, which account for the protection of salivary fluid secretion in MitoTEMPO-treated irradiated mice. Together, our findings identify ROS_mt_ as a primary driver of IR-induced salivary gland hypofunction. We suggest that MitoTEMPO treatment is a potentially important strategy for mitigating the deleterious effects of IR on salivary gland fluid secretion.

## Results

### MitoTEMPO treatment suppresses IR-induced initial increases in ROS_mt_, [Ca^2+^]_mt_, and [Ca^2+^]_i_ in mouse submandibular acinar cells

**Figure 1:**
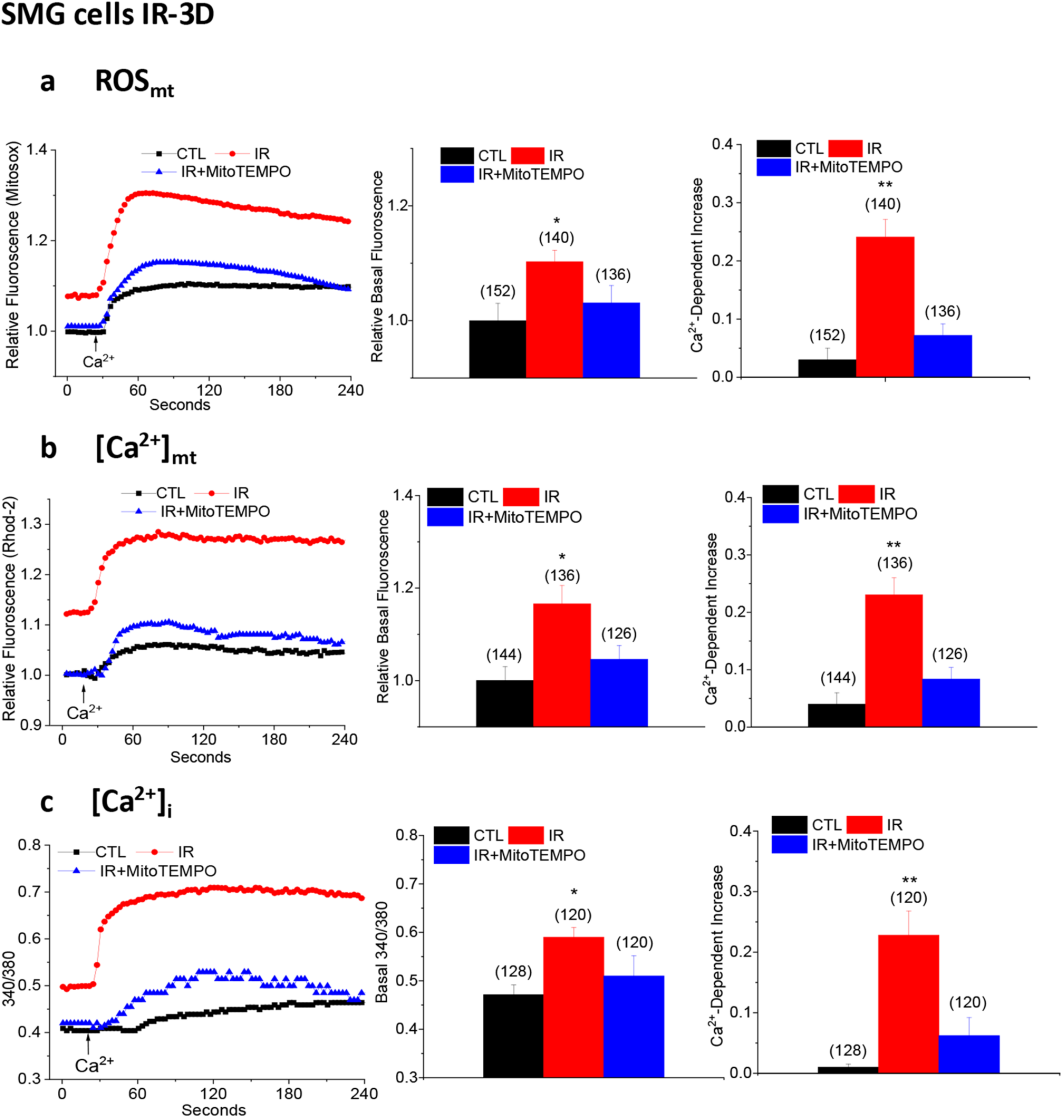
MitoTEMPO suppressed IR-induced increases in ROS_mt_, [Ca^2+^]_mt_, and [Ca^2+^]_i_ in submandibular acinar cells. (**a**) ROS_mt_ was measured by monitoring the fluorescence of ROS_mt_ indicator dye MitoSOX. Cells were first maintained in Ca^2+^-free medium to measure basal levels of ROS_mt_ (basal fluorescence). Ca^2+^ was then added to the external medium and Ca^2+^-dependent increase in fluorescence was measured. Traces in the left panel shows representative MitoSOX fluorescence changes in the three groups of cells, middle panel shows quantitation of data for basal ROS_mt_ and right panel shows Ca^2+^-dependent increase in ROS_mt_. Data were obtained from 132 to 152 cells from at least 3 different experiments. Values marked as *p < 0.05 and **p < 0.01 indicate significant difference as compared with CTL and IR + MitoTEMPO values (using unpaired *t* test). Unmarked values are not different from each other. (**b**) [Ca^2+^]_mt_ was monitored by measuring the Rhod-2 fluorescence in cells under Ca^2+^-free conditions and after Ca^2+^ was added to the external medium. Similar sets of cells were used as in (**a**). Traces are shown in left panel, and quantitation of the data are shown in the middle and right panels; basal [Ca^2+^]_mt_ and Ca^2+^-dependent increase in [Ca^2+^]_mt_, respectively. Data were obtained from 124–144 cells from at least 3 different experiments. Values marked as **p < 0.01 and *p < 0.05 indicate significant difference as compared to the CTL and IR + MitoTEMPO values. Unmarked values are not different from each other. Statistical comparisons were done using unpaired *t* test. (**c**) [Ca^2+^]_i_ was monitored by measuring the fura-2 fluorescence in cells maintained in Ca^2+^-free conditions and after Ca^2+^ was added to the external medium. Traces are shown in the in left panel, quantitation of data in middle and right panels are shown as basal changes of [Ca^2+^]_i_ and Ca^2+^-dependent increase in [Ca^2+^]_i_ after re-addition of external Ca^2+^. Data were obtained from 120–128 cells from at least 3 different experiments. Values marked as **p < 0.01 and *p < 0.05 are significantly different as compared with CTL and IR + MitoTEMPO. Unmarked values are not different from each other. Unpaired *t* test was used to determine statistical significance of data. Values shown are mean ± SEM in each case.

Radiation, single dose 15 Gy, was administered to the salivary gland region of mice with lead shielding to protect rest of the body as we have described earlier^13^. MitoTEMPO was given intraperitoneally to the mice as follows; first dose—5 mg/kg 10 min before irradiation; second dose—5 mg/kg 24 h after IR; and third dose—2.5 mg/kg 48 h after IR. This treatment protocol was based on our previous study^6^ which showed that ROS_mt_ in salivary gland acinar cells was highest 24–72 h after IR and then decreased to control levels by 30 days^6^. We assessed mitochondrial parameters; mitochondrial ROS (ROS_mt_, using MitoSOX), mitochondrial [Ca^2+^] ([Ca^2+^]_mt,_ using Rhod-2), and cytosolic [Ca^2+^] ([Ca^2+^]_i,_ using fura-2) in acinar cells of salivary glands obtained from mice 72 h post-irradiation after they have received all three doses of MitoTEMPO (control non-irradiated, CTL; irradiated, IR; and irradiated MitoTEMPO-treated, IR + MitoTEMPO). Basal ROS_mt_ in cells maintained in Ca^2+^-free medium was significantly higher in cells from IR mice compared to those from non-irradiated animals. Further, when Ca^2+^ was added to the external medium, there was a second increase in ROS_mt_ which was also higher in cells from IR mice compared to non-irradiated controls (Fig. 1a, compare red traces with black traces, also see average data in bar graphs shown in the middle and right panels). The increase in ROS_mt_ induced by addition of external Ca^2+^ represents the increased permeability of the plasma membrane to Ca^2+^ as a result of TRPM2 activation by the radiation treatment^6^. Importantly, both basal ROS_mt_ as well as Ca^2+^-dependent increase in acinar cell ROS_mt_ from MitoTEMPO-treated mice were not significantly higher than in cells from non-irradiated control mice. Thus, the MitoTEMPO treatment protocol was efficient in suppressing the initial increase in ROS_mt_ that is caused by IR.

As seen for ROS_mt_, resting [Ca^2+^]_mt_ and the additional increase induced by re-addition of Ca^2+^ to the external medium were both higher 3 days after IR in salivary gland acinar cells from irradiated mice as compared to those from non-irradiated control mice (Fig. 1b, compare red and black traces, also see bar graphs shown in the middle and right panels). Remarkably, these increases in [Ca^2+^]_mt_ (basal increase and Ca^2+^-dependent increase) in acinar cells from IR-mice receiving Mito-TEMPO were lower than IR-mice that did not receive MitoTEMPO (Fig. 1b, compare blue and red traces, also see bar graphs shown in the middle and right panels) and were not different when compared with the values in cells from control non-IR mice.

Ca^2+^ entering the cytosol via TRPM2 in irradiated cells is taken up into mitochondria causing an elevation in [Ca^2+^]_mt_ and ROS_mt_^6^. To examine whether TRPM2 function is affected we measured [Ca^2+^]_i_ in fura-2 loaded acinar cells from the same three sets of mice (non-IR control, IR, and IR + MitoTEMPO treated). Basal [Ca^2+^]_i_ as well as the increase detected upon re-addition of external Ca^2+^ were significantly higher in cells from IR mice as compared to those from control non-irradiated animals. Importantly, cells from IR + MitoTEMPO treated irradiated mice showed basal and external Ca^2+^-dependent [Ca^2+^]_i_ increase that were not significantly different from those in cells from non-irradiated mice (Fig. 1c, see traces and bar graphs).

We further confirmed the effect of MitoTEMPO by using HSG cells (Supplementary Fig. S1). Consistent with our previous findings^6^, IR caused increase in ROS_mt_, [Ca^2+^]_mt_, and [Ca^2+^]_i_ in HSG cells. Both basal and external Ca^2+^-dependent ROS_mt_ were higher in IR cells compared to non-IR controls. Similar to the findings described above in salivary gland acinar cells, MitoTEMPO treatment of HSG cells prior to IR significantly reduced IR-induced increases in ROS_mt_, [Ca^2+^]_mt_ and [Ca^2+^]_i_. However, in this case, values for ROS_mt_, [Ca^2+^]_mt_, and Ca^2+^-dependent increase in [Ca^2+^]_i_ in MitoTEMPO treated IR cells remained slightly higher than in the respective non-irradiated control cells.

Together the data in Fig. 1 demonstrate that treatment of mice with the mitochondrially targeted ROS scavenger, MitoTEMPO, prior to and immediately after radiation treatment, suppresses early changes in acinar cell mitochondria that are induced by IR, namely elevations in ROS_mt_ and [Ca^2+^]_mt_. Importantly, attenuation of IR-induced [Ca^2+^]_i_ increase by MitoTEMPO-treatment seen in Fig. 1c suggests that the increase in ROS_mt_ is a critical factor in the activation of plasma membrane Ca^2+^ influx via TRPM2 post-irradiation.

### MitoTEMPO treatment of mice suppresses IR-induced activation of capsase-3 and provides long-term protection of SOCE in salivary gland acinar cells

**Figure 2:**
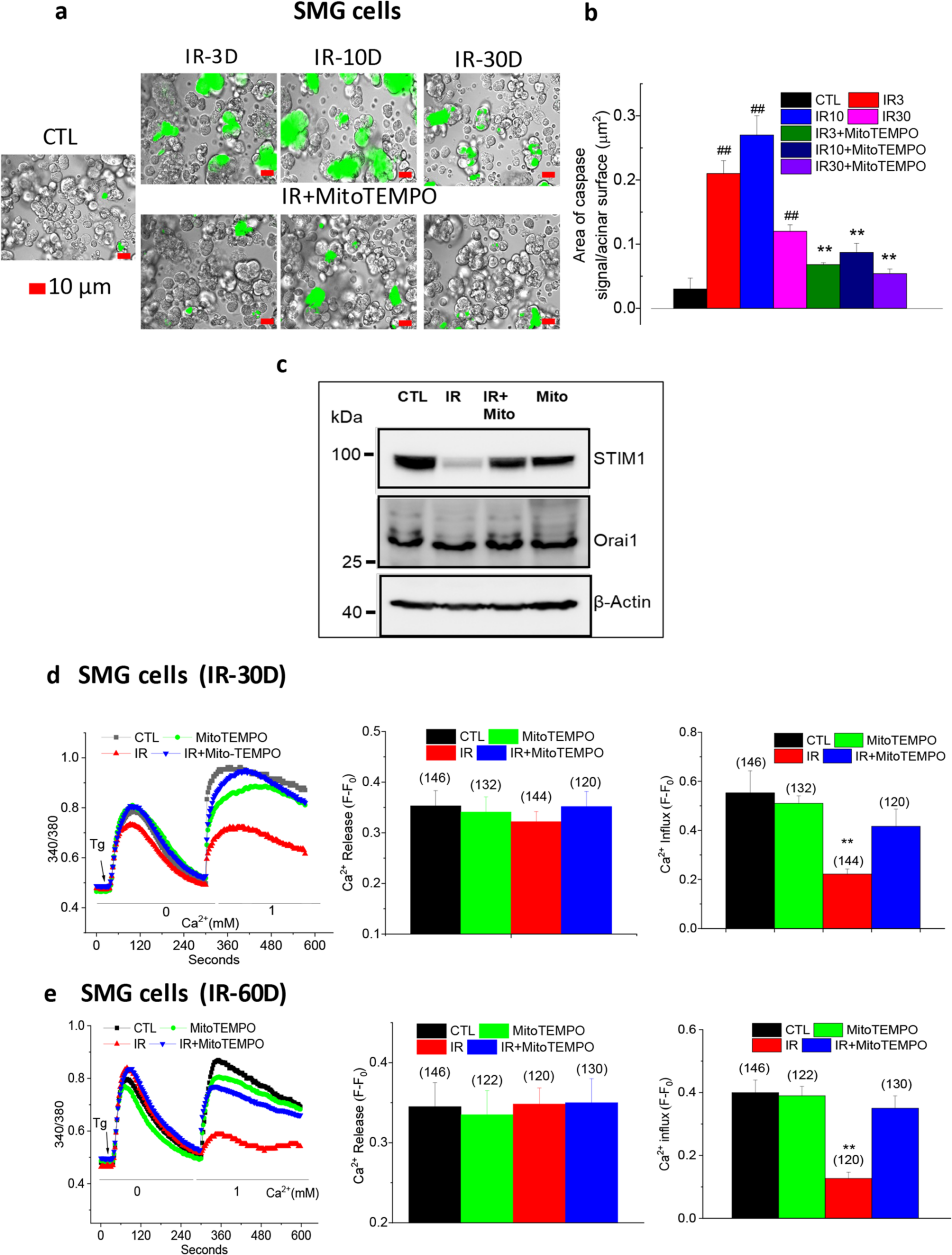
MitoTEMPO treatment reduced IR-dependent activation of capsase-3 and protected STIM1 and SOCE in salivary gland cells. (**a**) Activated caspase-3 was detected in submandibular acinar cells using CellEvent Caspase3/7 Green Detection Reagent. The groups of cells were IR (from mice receiving 15 Gy), IR + MitoTEMPO (from mice receiving IR and MitoTEMPO); and CTL (from non-irradiated control mice). Measurements were made on days 3, 10, and 30 after IR. (scale bar = 10 µm). (**b**) Quantitation of caspase-3 signal/total surface acinar area for various groups of cells shown in (**a**). Values marked ^##^p < 0.01, have been compared to CTL value; **p < 0.01, indicate values that are significantly different relative to IR group at the same time points. χ^2^ test was used for the statistical evaluation of the data. Data were analyzed from > 200 cells from at least 3 independent experiments. (**c**) Western blot showing amounts of STIM1 and Orai1 proteins in salivary gland samples from CTL, IR, IR + MitoTEMPO, and MitoTEMPO groups. Blot is representative of data obtained in 3 separate experiments. STIM1 is shown in upper blot, Orai1 in middle blot and actin as loading control in the lower blot (IB antibodies are indicated in the figure). STIM1 and Orai1 images are from the same blot. After the proteins were transferred onto the membrane, it was cut into two (between the 70 kDa and 50 kDa markers). The upper part of the membrane was probed for STIM1 whereas the lower part was probed for Orai1. For β-actin, the samples derived from the same experiment were used and all the steps (electrophoresis, transfer, blotting and imaging) were performed in parallel. Images of the uncropped blots for each protein are shown in supplemental section (Fig. S3). (**d**,**e**) Fura-2 fluorescence was monitored in acinar cells of mice 30 (**d**) and 60 (**e**) days after IR (CTL, MitoTEMPO, IR, and IR + MitoTEMPO as described above). SOCE was induced by Tg treatment. The left panels in c and d shows traces of fluorescence changes in the cells (representative of data from at least 3 individual experiments). The first increase in fluorescence in Ca^2+^-free medium represents Ca^2+^ release from ER and the second peak of fura-2 fluorescence indicates Ca^2+^ entry. Quantitation of Tg-induced Ca^2+^ release and Ca^2+^ influx are shown in middle and right panels, respectively. Data were obtained from 120 to 146 cells in at least 3 independent experiments in each case. **p < 0.01, indicates values that are significantly different as compared to unmarked values (calculate using unpaired *t* test). Values shown are mean ± SEM in each case.

Next, we examined the effect of MitoTEMPO treatment on IR-induced activation of caspase-3 in acinar cells isolated from salivary glands of mice; control non-irradiated, irradiated, and irradiated with MitoTEMPO treatment using Green Detection Reagent (CellEvent Caspase-3/7 Green Detection Reagent, Thermo Fisher Scientific). Submandibular gland acinar cells from irradiated mice had almost seven or ninefold increase in activated caspase-3 that was detected 3 and 10 days after IR respectively, which decreased to a fourfold increase by 30 days after IR (Fig. 2a, green fluorescence indicates activated cleaved caspase-3; average data and statistical analysis are shown in Fig. 2b). By comparison, caspase-3 detected in SMG acini from mice that were treated with MitoTEMPO was significantly less (about 75% lower) as compared with values obtained in IR-cells at the same time points but not different from that in control cells (Fig. 2a,b, the same sets of mice used for the experiments described in Fig. 1 were used here). Similar attenuation of IR-induced caspase-3 activation was seen in MitoTEMPO treated IR-HSG cells. In this case, caspase-3 activation was seen 6 h after IR (15 Gy) which increased up to 72 h after irradiation. In contrast, activated caspase-3 was not detectable in MitoTEMPO-treated irradiated cells to levels greater than in non-irradiated cells (Supplementary Fig. S2a).

Persistent defects in SOCE and STIM1 in submandibular gland acinar cells from mice that received irradiation to head and neck region can account for the loss of salivary gland fluid secretion^6^. The amounts of STIM1 and Orai1 in salivary gland samples from mice that received radiation either with or without MitoTEMPO treatment was examined. Western blots in Fig. 2c show a decrease in STIM1, but not Orai1, in samples from irradiated mice as compared with samples from control mice that did not receive IR or those that received only MitoTEMPO (samples were obtained from respective mouse salivary glands 60 days after IR). Importantly, this decrease in STIM1 was not detected in samples obtained from MitoTEMPO-treated irradiated mice (full images of blots provided in Supplemental Fig. 3). Based on our previous findings^13^, we can suggest that maintenance of STIM1 abundance in MitoTEMPO treated IR mice is associated with the suppression of caspase-3 activation. Further, enhancement of ROS_mt_ induced soon after IR underlies the more sustained caspase-3 activation as suppressing the former also prevented the latter.

**Figure 3:**
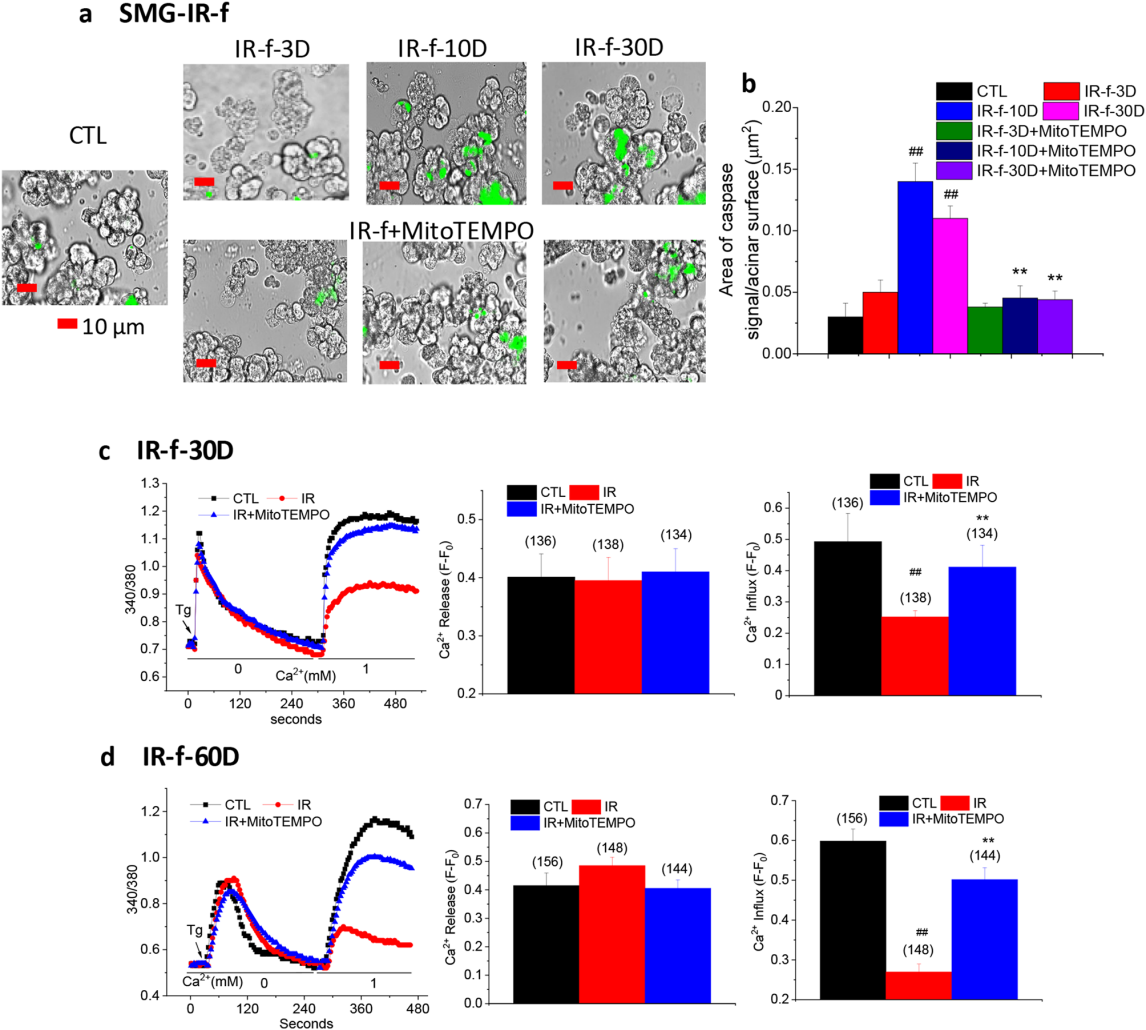
MitoTEMPO inhibited activation of capsase-3 and protected SOCE in mice receiving fractionated dose of IR. (**a**) Activated caspase-3 was detected in submandibular acinar cells using CellEvent Caspase3/7 Green Detection Reagent. SMG acinar cells were isolated from control mice (CTL) and those that received fractionated IR (IR-f) or IR + MitoTEMPO Cells were isolated 3, 10 and 30 days after IR. Representative data from at least 3 separate experiments are shown. Scale bar = 10 µm. (**b**) Bar graphs showing area of caspase-3 signal/total surface acinar area for each group of cells. χ^2^ test was used to determine the statistical significance. Values marked with **p < 0.01, indicate significant difference as compared with the same time point in the IR group, but not from CTL. ^##^p < 0.01, indicates values that are significantly different from CTL. Unmarked values are not different from each other. Data were obtained from > 200 cells per group from at least 3 independent experiments. (**c**,**d**) Fura-2 fluorescence was monitored in salivary gland acinar cells isolated from control mice (CTL), irradiated without MitoTEMPO (IR) and with MitoTEMPO treatment (IR + MitoTEMPO), 30 and 60 days after IR. Representative traces, left panels in c and d, show Tg-stimulated Ca^2+^ release (increase in 340/380 fluorescence ratio in Ca^2+^-free medium) and Ca^2+^ influx components (increase in 340/380 fluorescence ratio in when Ca^2+^ is added back to the medium). Average data are presented in the middle (peak change during release) and right panels (peak change during influx) in (**c**,**d**). The data were obtained from at least three different experiments (each with individual mice) with 134–156 cells for each group. Data shows mean ± SEM and statistical evaluation was done using unpaired *t* test. Unmarked values (middle panel) are not different from each other. Values marked ^##^ in bar graphs indicates significant difference as compared with CTL (p < 0.01); ** indicates values that are significantly different as compared with control and IR groups (p < 0.05).

Next, we measured SOCE in submandibular gland acinar cells isolated from control non-IR mice, MitoTEMPO-treated non-IR mice, IR mice, MitoTEMPO-treated IR mice. As can be predicted based on the decreased abundance of STIM1 protein in samples from IR mice, SOCE was reduced by about 60% at 30 and 68% at 60 days after IR as compared with the function in cells from control non-irradiated mice as well as MitoTEMPO-treated IR mice (CTL, MitoTEMPO, and IR + MitoTEMPO groups). In contrast, cells from IR + MitoTEMPO IR mice displayed a small decrease in SOCE which was not significant when compared to those from control mice (Fig. 2d,e) but was greater than SOCE in the irradiated group. Intracellular release induced by thapsgargin (Tg) was similar in all cells from all four sets of mice and not affected by IR or MitoTEMPO. Thus, IR-induced decrease of SOCE, a key requirement for neurotransmitter-stimulated salivary fluid secretion, was protected in salivary gland acinar cells obtained from MitoTEMPO-treated IR mice. Similar results were obtained in irradiated HSG cells. Irradiation-induced activation of caspase-3 as well as loss of SOCE were both prevented by MitoTEMPO treatment of cells immediately prior to delivery of a single dose of 15 Gy radiation (Supplementary Fig. S2a,b).

### MitoTEMPO suppresses activation of caspase-3 and protects SOCE in salivary gland cells from mice receiving fractionated dose of irradiation

We also investigated the effect of MitoTEMPO in mice subjected to fractionated doses of irradiation (IR-f). In this case, a total of 15 Gy was delivered to the head and neck region of mice over 5 day-period (each dose was 3 Gy delivered for 5 consecutive days). The mice received MitoTEMPO, 1 mg/kg IP injection, as follows: 1× prior to each 3 Gy IR (total for 5 days) and then 3× for 3 consecutive days after last IR. The lower dose of MitoTEMPO prior to each IR was based on the lower dose of IR (3 Gy instead of 15 Gy) and the frequency was based on ROS from our studies with single 15 Gy IR, showing it was highest soon after receiving IR. We examined activated caspase-3 and SOCE in salivary gland acinar cells from these mice, in control, (CTL, not irradiated and not treated with MitoTEMPO); irradiated (IR-f), and MitoTEMPO-treated irradiated (IR-f-MitoTEMPO) (Fig. 3). Control untreated mice were subject to sham IR treatment and injections. Activated caspase-3 was determined at 3, 10 and 30 days after last treatment with MitoTEMPO (i.e. 3 day time point is 6 days after last IR dose, 10 day time point is 13 days after last dose of IR). Interestingly, the time course of caspase-3 activation was somewhat different from that seen in cells from mice receiving a single dose of IR. On day 3 there was no increase in caspase 3 activity in cells from IR-f mice as compared with non-IR controls (Fig. 3a,b). In contrast, there is an almost sevenfold increase in activated caspase-3 3 days after a single dose of IR (see data in Fig. 2a). The level increased by day 10 and stayed quite elevated on day 30 after IR treatment. However, the maximum increase seen at the 10 days was 4.6-fold as compared with ninefold increase following single dose IR (see Fig. 2a). More importantly, MitoTEMPO treatment suppressed the IR-induced caspase-3 activation in the acinar cells from mice receiving fractionated dose IR. The level of caspase-3 activity in MitoTEMPO-treated cells, at 10 and 30 days, was reduced as compared with values at the same time points in IR cells. The values at the 3-day time point was similar in IR and IR + MitoTEMPO cells. All values obtained in MitoTEMPO-treated group were not different from that in control cells. These findings suggest that the effect of subjecting mice to fractionated IR might be slower and less severe than that produced by a single dose. It is possible that in IR-f, the effects are likely to be cumulative with time for potential recovery mechanisms to be elicited within the 24-h time intervals between successive doses. Also, of significance is that activated caspase-3 is more persistent in this case. However, it is important to appreciate that the amplitude and time-course of these changes would depend on the dose of IR and length of the treatment.

As can be predicted based on suppression of caspase activity, MitoTEMPO treatment also induced protection of SOCE measured 30 and 60 days after the mice received fractionated IR. SOCE in acinar cells from irradiated mice was significantly lower by about 60%, as compared with cells from control non-irradiated mice as well as from MitoTEMPO-treated mice that were not irradiated. Importantly, SOCE in cells from MitoTEMPO-treated irradiated mice (Fig. 3c,d) was significantly higher than that in IR group and a bit lower than in CTL and MitoTEMPO-treated CTL mice. Tg-stimulated intracellular release was not affected by IR or MitoTEMPO treatment.

### MitoTEMPO treatment protects against loss of salivary gland fluid secretion in irradiated mice

**Figure 4:**
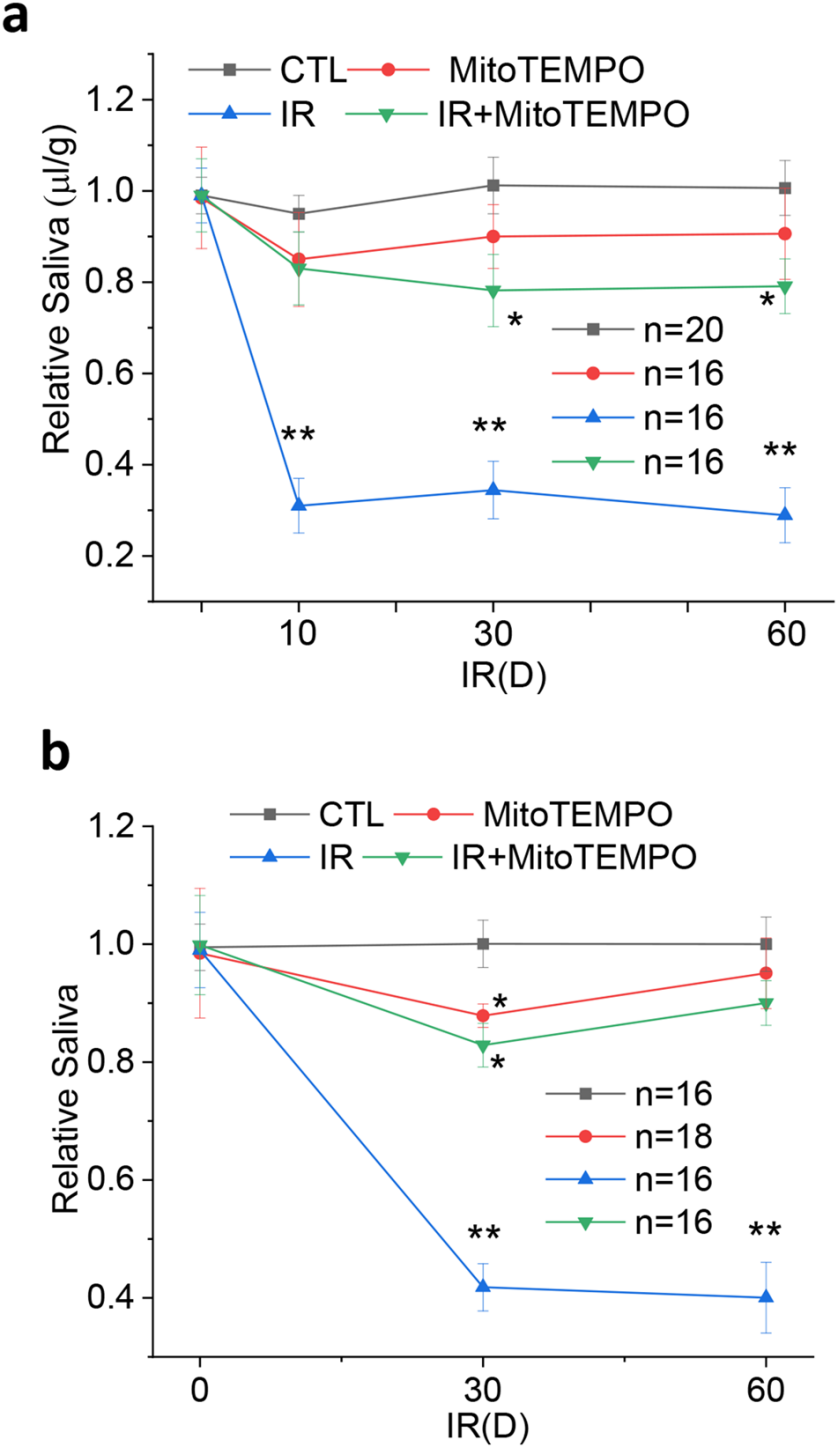
MitoTEMPO rescued the loss of salivary fluid secretion in irradiated mice. (**a**) Pilocarpine-stimulated salivary fluid secretion was monitored in control mice (CTL), irradiated mice (IR), and mice receiving MitoTEMPO with or without IR, on day 0 (24 h prior to IR) and days 10, 30 and 60 after IR. MitoTEMPO was given at 5 mg/kg i.p. 10 min before IR, 24 h after irradiation, followed by a 2.5 mg/kg 48 h after IR. 16 to 20 mice were used as indicated and data are presented as mean ± SEM. *P < 0.05, indicated values that are significantly different from CTL values at the same time point and **P < 0.01, indicate values that are significantly different as compared to control and MitoTEMPO treated (no IR) at the same time points (unpaired *t* test). (**b**) Pilocarpine-stimulated salivary fluid secretion was monitored in the same groups of mice; CTL, MitoTEMPO, IR, IR + MitoTEMPO on day 0 (24 h before IR) and on days 30 and 60 after IR. In this case mice were delivered fractionated IR (IR-f, 3 Gy per day for 5 consecutive days) MitoTEMPO was administered at a dose of 1 mg/kg i.p. 10 min before each IR for 5 days and then repeated for every 24 h for 3 more days. 16 to 18 mice were used as indicated. Mean ± SEM, *P < 0.05, indicated values that are significantly different from CTL values at the same time point and **P < 0.01, indicate values that are significantly different from all other groups values at the same time point (unpaired *t* test).

Figure 4a shows the effect of MitoTEMPO treatment on IR-induced loss of salivary gland function in mice receiving single dose of IR. 10 days after single 15 Gy IR, control mice displayed 70% loss of saliva secretion and this decrease was maintained up to 60 days (longest time point measured in this study). In contrast, MitoTEMPO-treated mice that received IR lost only 20% of the function and retained 80% of fluid secretion 30 and 60 days after irradiation. On day 10 after IR treatment, the MitoTEMPO group displayed almost 90% of the function, the small 10% decrease was not significant when compared with the control group.

An important finding in this study is that MitoTEMPO was also effective in protecting salivary gland function in mice receiving fractionated dose of IR (Fig. 4b). Pilocarpine-stimulated saliva was measured on days 30 and 60 after IR. Salivary secretion was down about 60% after 30 and 60 days after irradiation, about 10% less reduction as compared with that in mice receiving single dose of 15 Gy. Importantly, those receiving MitoTEMPO and IR-f displayed almost twofold higher levels of saliva secretion as compared with the IR group. However, in this set of experiments, mice treated with MitoTEMPO alone or MitoTEMPO with IR-f both displayed a small, but significant decrease in saliva secretion on day 30, but not day 60, as compared with the non-irradiated group (the MitoTEMPO and IR-f-MitoTEMPO groups were not significantly different from each other). This might reflect the higher number of doses and total amount of MitoTEMPO received by this group of mice. Importantly, the small decrease in fluid secretion at day 30 after IR was reversible and mice regained normal levels of secretion by 60 days. Together, these data suggest that an important outcome of MitoTEMPO treatment is the protection of salivary gland function at the early time points, i.e. 10 days after IR.

## Discussion

Reactive oxygen species are regarded as crucial players in radiation-induced salivary gland tissue injury. In vivo and in vitro experiments showed that ROS levels sharply increase in salivary glands after IR^1,14^. Such increases in ROS have been suggested to induce DNA damage, inhibit cell proliferation, and damage potential resident stem cells that could differentiate into acinar cells^14^. Our previous studies have shown that IR of salivary glands causes early effects on mitochondria, including increases in [Ca^2+^]_mt_, ROS_mt_, and loss of membrane potential. Further, the changes in mitochondria induce activation of caspase-3, which lead to decrease in the ER protein STIM1 and consequently, attenuation of SOCE, in acinar cells. The latter can account for IR-induced salivary gland dysfunction as it provides the necessary increase in [Ca^2+^]_i_ required to drive secretion of fluid from the gland^2,6,13^. Together, our previous data demonstrated that mitochondria are early responders and central players in IR-induced salivary gland dysfunction. Herein we report that suppression of the initial increase in mitochondrial ROS elicits significant protection of salivary gland secretion in irradiated mice. Based on findings obtained with mice that were treated with mitochondrially targeted ROS scavenger MitoTEMPO and subjected to either a single or fractionated dose of IR we suggest that the initial increase in ROS_mt_ is a critical factor that drives salivary gland dysfunction following irradiation.

Our findings demonstrate that salivary gland cells from mice that were treated with MitoTEMPO prior to and immediately after IR display attenuation of initial radiation-induced increase in ROS_mt_. Our previous study demonstrated a significant increase in ROS_mt_ 1 day after irradiation and further increases by days 3 and by 10. Thereafter it decreases to almost resting levels by 30 Days of IR^6^. By treating mice with the ROS scavenger within this time period after IR (days 1–3), we find that cells from MitoTEMPO-treated mice display ROS_mt_ that is about 80% lower than in cells from untreated, IR mice. Thus, MitoTEMPO is an effective suppressor of irradiation induced elevation of ROS_mt_. In addition, scavenging ROS_mt_ also suppressed IR-induced increase in [Ca^2+^]_mt_. Under physiological conditions, [Ca^2+^]_mt_ is beneficial for mitochondrial function. However, elevations of [Ca^2+^]_mt_ can trigger increases in ROS_mt_. Furthermore, the presence of an overriding pathological stimulus [Ca^2+^]_mt_ can be detrimental as it can also enhance ROS_mt_. Thus, IR can cause enhancement of ROS_mt_ directly and indirectly via triggering an increase in [Ca^2+^]_mt_^6^. Our previous data had shown that TRPM2 activation by IR leads to an influx of Ca^2+^ into the cytosol with subsequent uptake into the mitochondria^13^. Here we show that both the increase in [Ca^2+^]_i_, due to TRPM2 activity, and elevation of [Ca^2+^]_mt_, due to uptake from the cytosol, are attenuated in cells from IR + MitoTEMPO-treated mice. These data further corroborate our suggestion that IR-induced increase ROS_mt_ is critical in the activation of TRPM2. ADP ribose, the intracellular activator of TRPM2, is increased by IR^6,13^. Further, ROS_mt_ also contributes to this increase in ADPR^6^. Thus, when the latter is clamped by MitoTEMPO, the feedforward loop which leads to TRPM2 activation is also attenuated. Finally, we show that activation of caspase-3 which is more delayed than the increase in ROS_mt_ and [Ca^2+^]_mt_ (present study) and loss of membrane potential^6,13^, is also suppressed by MitoTEMPO treatment. These findings provide strong evidence that key early changes in salivary gland acinar cells that are induced by irradiation are effectively prevented by suppressing IR-induced elevation of ROS_mt_ by MitoTEMPO treatment. As a result, loss of STIM1 protein and SOCE are prevented and salivary gland function is protected.

MitoTEMPO has received much attention recently due to its effect in ameliorating several conditions of ROS-induced cellular dysfunction and damage^15,16^. It has been used to protect against ROS-associated disorders, such as oxalate induced kidney injury as well as urolithiasis, heart failure, and sudden cardiac death^17,18^. It is also reported to improve cardiovascular performance in older patients^19^. MitoTEMPO pretreatment enhanced the mitochondrial antioxidant capacity of adipose-derived stem cells^20^. Interestingly, intranasal delivery of MitoTEMPO, ROS_mt_ antioxidant, alleviate influenza virus pathology^21^ due to its ability of preventing the increase in ROS_mt_. While this represents the first report describing the effects of MitoTEMPO on IR-induced salivary gland dysfunction, we had reported earlier that treatment of mice with the stable nitroxyl antioxidant, TEMPOL, which predominantly scavenges ROS from the cytoplasm^22^, induces significant protection of salivary fluid secretion in IR mice, 70% function was retained 30 and 60 days after IR^13^. However, TEMPOL-treated mice still displayed 50% decrease in saliva flow at the 10-day time point as compared to the < 10% decrease in function seen in MitoTEMPO treated mice. Based on these previous and the present findings we suggest that scavenger of mitochondrial ROS elicits greater protection of salivary gland function in irradiated mice than a cytosolic ROS scavenger. More importantly, the protective effect exerted by MitoTEMPO is detected immediately after IR. In aggregate, our findings demonstrate that with MitoTEMPO treatment there is minimal decrease in salivary gland function following IR. Future studies are needed to determine whether MitoTEMPO also protects other tissues, such as nerves and blood vessels, within the salivary gland.

In conclusion, a severe consequence of radiation therapy in patients with head and neck cancer is persistent salivary gland hypofunction which causes xerostomia and oral infections. We previously showed that irradiation (IR) of salivary glands in mice triggers initial transient increases in ROS_mt_, [Ca^2+^]_mt_ and caspase-3 activation but persistent loss of salivary secretion. Herein we assessed the role of ROS_mt_ in radiation-induced irreversible loss of salivary gland function. We report here that treatment of mice with the mitochondrially-targeted antioxidant, MitoTEMPO, resulted in almost complete protection of salivary gland secretion following either single (15 Gy), or fractionated (5 × 3 Gy), doses of irradiation. Salivary gland cells isolated from MitoTEMPO-treated, irradiated, mice displayed significant attenuation of the initial increases in ROS_mt_, [Ca^2+^]_mt_ and activated caspase-3 as compared to cells from irradiated, but untreated, animals. Importantly, MitoTEMPO treatment prevented radiation-induced decrease in STIM1, consequently protecting store-operated calcium entry which is essential for saliva secretion. Together, these findings identify initial increase in ROS_mt_ induced by irradiation as a critical driver of persistent salivary gland hypofunction. We suggest that the mitochondrially targeted antioxidant, MitoTEMPO, can be potentially important strategy for preventing salivary gland dysfunction that is induced by irradiation.

## Materials and Methods

### Animals and cell culture

SMG acinar cells were isolated as described earlier^6^ from mouse glands by digesting with enzyme Liberase TL (Roche Diagnostics). Mice were euthanized by CO_2_ asphyxiation and the salivary glands were immediately excised. The dissected glands were finely minced and digested in Eagle’s minimum essential medium for 25 min in the presence of Liberase TL (0.5 mg/ml). After centrifugation, the cell pellet was further digested with Liberase TL for another 25 min and finally resuspended in Eagle’s minimum essential medium. Cells were continuously gassed with a mixture of 95% O_2_ and 5% CO_2_. Mice breeding and all other animal procedures were carried out using a protocol approved by the Animal Care and Use Committee, National Institute of Craniofacial Research, NIH, in compliance with the Guide for the Care and Use of Laboratory Animal Resources, National Research Council. This study was carried out in compliance with the ARRIVE guidelines. HSG cells were cultured on glass coverslips with Earle’s minimum essential medium (EMEM). EMEM was supplemented with 10% fetal calf serum, 2 mM glutamine, and 1% penicillin/streptomycin and cells were cultured at 37 °C in 5% CO_2_.

### Irradiation of mice and HSG cells

A single radiation dose of 15 Gy was delivered to the animal as described earlier^6^ by a Therapax DXT300 X-ray irradiator (Pantak Inc.) using 2.0-mm Al filtration (300-kV peak) at a dose rate of 1.9 Gy/min. Fractional radiation was carried out by irradiating mice with 3 Gy dose daily for 5 days. Immediately after irradiation, the animals were removed from the Lucite jig and housed (five animals per cage) in a climate- and a light/dark-controlled environment and allowed free access to dough diet (soft food). Salivary gland irradiation was accomplished by placing each animal into a specially built Lucite jig such that the animal could be immobilized without the use of anesthetics. In addition, the jig was fitted with a Lucite cone that surrounded the head and prevented head movement during the radiation exposure^6,13^. Lead shields were designed to cover the jigs with the mice with a small aperture in the lead shield that allowed radiation to the salivary gland area of the immobilized animal. HSG cells were cultured in glass-bottom dishes and irradiated in a Gamma cell 1000 irradiator with a single dose of radiation or using x-ray as described previously^13^.

### MitoTEMPO administration

For single dose of radiation experiments, mice received three IP injections of MitoTEMPO. First two doses were of 5 mg/kg (first dose, 10 min before radiation and second dose, 24 h after radiation) and the third dose was at a half of the initial dose (2.5 mg/kg) 48 h after radiation. Same treatment protocol was followed for MitoTEMPO control mice without radiation. For fractional radiation protocol mice were administrated with eight IP injections of MitoTEMPO (1 mg/kg each). The first five 1× doses were injected 10 min before each of the first five radiations of 3 Gy and the last three doses (3×) were injected every day for three days after last dose of radiation.

### Saliva secretion measurements

Salivary secretion was measured in control and irradiated mice 30 and 60 days after irradiation. For whole saliva collection, anesthetized mice were treated with pilocarpine solution (0.25 mg/kg) and the saliva was collected as described earlier^6^.

### [Ca^2+^]_i_ measurements

[Ca^2+^]_i_ in HSG cells or acini from mouse SMG were measured in Fura-2–loaded cells using an Olympus × 51 microscope (Olympus Centre Valley), with an ORCA-ER camera (Hamamatsu) attached to a Polychrome V (Till Photonics LLC). Cells were excited at 340/380 nm with an emission of 510 nm. MetaFluor (Molecular Devices) was used to acquire images and process data.

### [Ca^2+^]_mt_ measurement

[Ca^2+^]_mt_ measurement in HSG cells or acini from mouse SMG were done as described earlier^6^. Briefly, cells were loaded with 1 μM Rhod2/acetoxymethyl for 30 min and washed three times with SES (standard external solution) for 15 min at 37 °C. Cells were excited at 550 nm with an emission of 580 nm. Mitochondrial Ca^2+^ concentrations were expressed as normalized fluorescence ratio (*F*/*F*_0_, where *F*_0_ is the baseline fluorescence).

### Mitochondrial ROS measurement

HSG cells seeded on glass coverslips or acini from mouse SMGs were loaded with the mitochondrial O_2_-specific fluorescent probe MitoSOX (1 μM) for 10 min at 37 °C. MitoSOX fluorescence was analyzed using Polychrome V using 510 nm excitation wavelength^6^. The relative fluorescence intensity was used to indicate mitochondrial ROS as *F*/*F*_0_ (MitoSOX).

### Detection of cleaved caspase-3 in HSG and acinar cells

Active caspase-3 was detected in HSG cells and isolated submandibular acini as described earlier^6^ using the CellEvent Caspase-3/7 Green Detection Reagent (Thermo Fisher Scientific) with an absorption/emission maximum of 502/530 nm. HSG cells and isolated submandibular acini were plated on glass coverslips and incubated with the reagent at 1 μM concentration for 30 min. The reagent was detected using an Olympus × 51 microscope (Olympus), with an ORCA-ER camera (Hamamatsu) attached to a Polychrome V (Till Photonics LLC). MetaFluor (Molecular Devices) was used to acquire images and process data as described above. Apoptotic cells with activated caspase3/7 have bright green nuclei, whereas cells without activated caspase-3 have minimal fluorescence signal.

### Western blotting

Freshly excised mouse salivary glands were minced and lysed in RIPA buffer containing protease inhibitors. The protein lysates were mixed with tris–glycine sodium dodecyl sulfate sample buffer (Invitrogen) and loaded onto gels for Western blot analysis. Rabbit monoclonal anti-STIM1(1:1000 dilution, Cell Signaling Technologies, Danvers, MA), anti-Orail1 antibody (1:1000 dilution, custom generated^23^), and mouse polyclonal anti–β-actin antibody (1:5000 dilution, Abcam) were used for immunoblotting. After incubation with secondary antibodies, signals were detected by chemiluminescence using SuperSignal West Femto Maximum Sensitivity Substrate (Thermo Scientific) and ChemiDoc imaging system (Bio-Rad).

### Statistics

Data analyses were performed using Origin 9.0 (OriginLab). Statistical significance was determined using unpaired *t* test between two groups, whereas comparisons of multiple groups were made using ANOVA, followed by Sidak multiple comparisons test and the χ^2^ test for responses in cell populations. Data are expressed as means ± SE. Differences in the mean values were significant at * or ^#^*P* < 0.05 and ** or ^##^p < 0.01.

## Supplementary Information

Supplementary Information.

## References

[1] Ambudkar I (1865). Calcium signaling defects underlying salivary gland dysfunction. Biochim. Biophys. Acta Mol. Cell Res..

[2] Ambudkar IS (2016). Calcium signalling in salivary gland physiology and dysfunction. J. Physiol..

[3] Melvin JE, Yule D, Shuttleworth T, Begenisich T (2005). Regulation of fluid and electrolyte secretion in salivary gland acinar cells. Annu. Rev. Physiol..

[4] Teos LY (2016). Adenovirus-mediated hAQP1 expression in irradiated mouse salivary glands causes recovery of saliva secretion by enhancing acinar cell volume decrease. Gene Ther..

[5] Zeng M (2017). Restoration of CFTR activity in ducts rescues acinar cell function and reduces inflammation in pancreatic and salivary glands of mice. Gastroenterology.

[6] Liu X (2017). Radiation inhibits salivary gland function by promoting STIM1 cleavage by caspase-3 and loss of SOCE through a TRPM2-dependent pathway. Sci. Signal.

[7] Jensen AI (2020). A solid support generator of the Auger electron emitter rhodium-103m from [(103)Pd]palladium. Appl. Radiat. Isot.

[8] Langendijk JA (2008). Impact of late treatment-related toxicity on quality of life among patients with head and neck cancer treated with radiotherapy. J. Clin. Oncol..

[9] Liu X, Ong HL, Ambudkar I (2018). TRP channel involvement in salivary glands-some good. Some Bad. Cells.

[10] Wu VWC, Leung KY (2019). A review on the assessment of radiation induced salivary gland damage after radiotherapy. Front. Oncol..

[11] Khodamoradi E (2020). Targets for protection and mitigation of radiation injury. Cell Mol. Life Sci..

[12] Sun Y (2020). ROS systems are a new integrated network for sensing homeostasis and alarming stresses in organelle metabolic processes. Redox Biol..

[13] Liu X (2013). Loss of TRPM2 function protects against irradiation-induced salivary gland dysfunction. Nat. Commun..

[14] Tateishi Y, Sasabe E, Ueta E, Yamamoto T (2008). Ionizing irradiation induces apoptotic damage of salivary gland acinar cells via NADPH oxidase 1-dependent superoxide generation. Biochem. Biophys. Res. Commun..

[15] Ding W, Liu T, Bi X, Zhang Z (2017). Mitochondria-targeted antioxidant mito-tempo protects against aldosterone-induced renal injury in vivo. Cell Physiol. Biochem..

[16] Olgar Y (2018). Aging related functional and structural changes in the heart and aorta: MitoTEMPO improves aged-cardiovascular performance. Exp. Gerontol..

[17] Dey S, DeMazumder D, Sidor A, Foster DB, O'Rourke B (2018). Mitochondrial ROS drive sudden cardiac death and chronic proteome remodeling in heart failure. Circ. Res..

[18] Zhang J (2017). MitoTEMPO prevents oxalate induced injury in NRK-52E cells via inhibiting mitochondrial dysfunction and modulating oxidative stress. Oxid. Med. Cell Longev..

[19] Olgar Y, Tuncay E, Turan B (2019). Mitochondria-targeting antioxidant provides cardioprotection through regulation of cytosolic and mitochondrial Zn(2+) levels with Re-distribution of Zn(2+)-transporters in aged rat cardiomyocytes. Int. J. Mol. Sci..

[20] Lian K (2019). Pretreatment of diabetic adipose-derived stem cells with mitoTEMPO reverses their defective proangiogenic function in diabetic mice with critical limb ischemia. Cell Transplant..

[21] To EE (2020). Mitochondrial reactive oxygen species contribute to pathological inflammation during influenza A virus infection in mice. Antioxid. Redox Signal..

[22] Wilcox CS, Pearlman A (2008). Chemistry and antihypertensive effects of tempol and other nitroxides. Pharmacol. Rev..

[23] Subedi KP, Ong HL, Son GY, Liu X, Ambudkar IS (2018). STIM2 induces activated conformation of STIM1 to control Orai1 function in ER-PM junctions. Cell Rep..

