# Author Correction: Mitochondria-targeted antioxidant protects against irradiation-induced salivary gland hypofunction

**DOI:** 10.1038/s41598-021-94087-7

**Published:** 2021-07-12

**Authors:** Xibao Liu, Krishna P. Subedi, Changyu Zheng, Indu Ambudkar

**Affiliations:** 1 Secretory Physiology Section, National Institute of Dental and Craniofacial Research, NIH, 9000 Rockville Pike, Bethesda, MD 20892 USA; 2 NIH, Building 10, Room 1N‑113, Bethesda, MD 20892 USA

Correction to: *Scientific Reports* https://doi.org/10.1038/s41598-021-86927-3, published online 08 April 2021

The Supplementary Information file published with this Article contained an error, where the Supplementary Figures S1, S2 and S3 were omitted.

The original Supplementary Information file is provided below.

The Supplementary Information file that accompanies the original Article has been corrected.

## Supplementary Information

Supplementary Information.

