# Supplementary material for: Mitochondria-targeted antioxidant protects against irradiation-induced salivary gland hypofunction
Source: Sci Rep. 2021 Apr 8;11:7690. doi: 10.1038/s41598-021-86927-3 (PMC8032724; doi:10.1038/s41598-021-86927-3)
Supplement: Supplementary file 1 — Supplementary Information. [file 41598_2021_86927_MOESM1_ESM.pdf]

Supplemental Figure legends and Supplemental Figures

Mitochondria-targeted Antioxidant Protects Against Irradiation-Induced Salivary Gland Hypofunction

Xibao Liu, Krishna P. Subedi, Changyu Zheng, and Indu Ambudkar*

Secretory Physiology Section, National Institute of Dental and Craniofacial Research,

NIH, 9000 Rockville Pike, Bethesda, Maryland 20892, USA

*Correspondence and requests for materials should be addressed to I.A.

Indu S. Ambudkar
Building 10, Room 1N-113
NIH, Bethesda MD, 20892.
Phone: 301-496-5298
Email: indu.ambudkar@nih.gov

Supplemental Figure Legends

Supplemental Fig. S1. MitoTEMPO reduced IR-induced increase in mitochondrial ROS, $[Ca^{2+}]_{mt}$ and $[Ca^{2+}]_i$ in HSG cells. **(a)** Mitochondrial ROS (ROS_{mt}) was monitored in control (CTL), irradiated (IR) and IR+MitoTEMPO (0.1μM MitoTEMPO, 10 minutes prior to IR treatment) treated HSG cells. Representative fluorescence traces are shown in the left panel and quantitation of data (mean±SEM) for basal ROS_{mt} (detected in Ca^{2+}-free medium) and Ca^{2+}-dependent increase in ROS_{mt} are shown in middle and right panels (data were obtained from 128-146 cells from multiple independent experiments). **, $p<0.01$, indicates values that are significantly different as compared to its respective control and IR+MitoTEMPO groups, $^{\#\#}$, $p<0.01$, indicates values that are significantly different as compared to its respective control using unpaired *t* test. **(b)** $[Ca^{2+}]_{mt}$ in the same groups of cells described in "a" was monitored by measuring Rhod-2 fluorescence in cells under Ca^{2+}-free conditions and after Ca^{2+} was added to the external medium (representative fluorescence traces are shown in the left panel). Quantitation of data, means ± SEM, are shown in bar graphs, middle and right panels are for basal $[Ca^{2+}]_{mt}$ under Ca^{2+}-free medium and peak Ca^{2+}-dependent increase in $[Ca^{2+}]_{mt}$, respectively (142-160 cells were used in at least 3 separate experiments). **, $p<0.01$, indicates values that are significantly different as compared to its respective both control and IR+MitoTEMPO groups. $^{\#\#}$, $p<0.01$, indicates values that are significantly different as compared to its respective control using unpaired *t* test. **(c)** $[Ca^{2+}]_i$ was monitored by measuring fura-2 fluorescence in cells maintained in Ca^{2+}-free conditions and after Ca^{2+} was added to the external medium in left panel. Quantitation of data shown in middle and right panels are for the basal changes of $[Ca^{2+}]_i$ under Ca^{2+}-free medium and peak increase in $[Ca^{2+}]_i$ after re-addition of external Ca^{2+}, respectively (119-145 cells from

multiple independent experiments). *, $p<0.05$ or **, $p<0.01$, indicates values that are significantly different as compared to its respective both control and IR+MitoTEMPO groups, ##, $p<0.01$, indicates values that are significantly different as compared to its respective control using unpaired *t* test. Data shown are mean ± SEM.

Supplemental Fig. S2. Effect of MitoTEMPO on irradiation induced caspase-3 activation and SOCE in HSG cells. **(a)** Detection of caspase-3 activation using the CellEvent Caspase3/7 Green Detection Reagents in IR-cells (15 Gy) treated or not with MitoTEMPO in HSG cells at different time from 6 to 72 hours after irradiation with activated caspase-3 (indicated by green fluorescence signal) in control or IR-cells treated with or without MitoTEMPO. χ^2 test was used to determine the statistical significance. Values marked with **, $p<0.01$, indicate statistically significant difference as compared to the same time point in the IR group, but not from CTL. ##, $p<0.01$, indicates values that are significantly different from CTL. The scale bar indicated 10 μm. Data were obtained from >200 cells from multiple independent experiments. **(b)** Fura-2 fluorescence ratio (340/380, reflecting $[Ca^{2+}]_i$) was monitored in control and irradiated HSG cells with or without treatment of MitoTEMPO. Representative fluorescence traces in left panel show Tg-induced intracellular Ca^{2+} release and Ca^{2+} influx. Bar graphs in middle and right panels show quantitation of the data (mean ± SEM) obtained from 116 to 126 cells in at least 3 independent experiments. Values marked **, $p<0.01$, are significantly different from the unmarked values (unpaired *t* test) compared to irradiated cells to its control and MitoTEMPO-treated cells. Data shown are mean ± SEM.

Supplemental Fig. S3. Images of full blots, with multiple exposures, which were cropped for the Western blot data shown in Fig. 2c. Amounts of STIM1, Orai, and β-actin in salivary gland samples from CTL, IR, IR+MitoTEMPO, and MitoTEMPO groups are shown (1,2,3,4). First three images in each case are chemiluminescent images of the protein bands acquired using ChemiDoc imager using different exposure times for the three proteins. The fourth set of images are the colorimetric images of the same membranes. The black lines on the colorimetric membrane images (at either end of the blot) show the position of molecular weight markers. Superimposed images of colorimetric and chemiluminescent images are shown in the fifth row. STIM1 and Orai1 proteins were detected using the same blot. After the protein transfer, the membrane was cut into two (between the 70kDa and 50 kDA markers), The upper part of the membrane was probed for STIM1 whereas the lower part was probed for Orai1. For β-actin, the samples derived from the same experiment were used and all the steps (electrophoresis, transfer, blotting and imaging) were performed in parallel. Anti-STIM1 and anti-actin antibodies are very sensitive and thus relatively short exposure times were required to detect the proteins. Colorimetric images used to detect the molecular weight markers show the edge of the paper for the STIM1 and actin blots. The chemiluminescence and colorimetric images were superimposed to align the protein bands with the molecular weight markers.
